# Navigating Anesthetic Management in the Setting of Uterine Rupture and Spinal Fracture: A Case Report

**DOI:** 10.7759/cureus.108717

**Published:** 2026-05-12

**Authors:** Lilly P Deljoo, Emily Major, Stewart Carter, Justin Jatczak

**Affiliations:** 1 Obstetrics and Gynecology, University of Louisville School of Medicine, Louisville, USA; 2 Pediatric Surgery, University of Louisville School of Medicine, Louisville, USA

**Keywords:** logrolling maneuver, motor vehicle accident, obstetric trauma, spinal instability, uterine rupture

## Abstract

Intrapartum trauma with spinal instability may require delivery by cesarean section (CS). While regional anesthesia is typically preferred in CS, general anesthesia may be necessary in emergent presentations that require rapid induction of anesthesia. We report a case wherein a patient presented with uterine rupture and spinal instability following a motor vehicle accident and received regional anesthesia prior to CS. A 27-year-old multiparous female at 37 + 6 weeks of gestation presented to the Emergency Department after a high-speed motor vehicle accident with significant abdominal pain and contractions, and a positive seatbelt sign was noted on examination. CT imaging revealed multiple spinal fractures and an apical pneumothorax. A spinal block was placed with the patient in the lateral decubitus position, after which she was logrolled to the dorsal position for surgery. A cesarean section was then performed, resulting in the delivery of a viable female infant. This case emphasizes the need for comprehensive clinical assessment when administering anesthesia in obstetrical trauma with spinal instability. Obstetricians and gynecologists are uniquely positioned within the multidisciplinary team to advocate for optimal maternal and fetal outcomes.

## Introduction

Pregnant patients are especially vulnerable to blunt abdominal trauma, which may result in obstetrical emergencies such as placental abruption and uterine rupture. Though exceedingly rare, uterine rupture is an obstetrical emergency that most commonly occurs in patients with scarred uteri from prior uterine surgeries; however, it may also occur in patients with an unscarred uterus or nulliparous patients [1-3]. Uterine rupture in unscarred uteri carries a greater risk for mortality compared to scarred uteri, with an estimated perinatal death rate of 12-35% and a maternal hysterectomy rate of 20-31% [4].

The American Society of Anesthesiologists Task Force on Obstetric Anesthesia’s updated recommendations state that neuraxial techniques are preferred relative to general anesthesia, but in cases of obstetrical emergency, including uterine rupture, general anesthesia may be preferred [5]. Additionally, each patient should be assessed as an individual and consider a comprehensive picture, including history and physical exam, intrapartum platelet count, blood type and screen, and perianesthetic fetal heart rate patterns. These guidelines do not address cases of spinal instability, and, therefore, further consultation with neurosurgery teams may be necessary. 

Here, we present a case of a midline uterine rupture occurring in the third trimester following a high-speed motor vehicle accident. A multidisciplinary team, including anesthesiology and neurosurgery, quickly assessed the patient and determined she was a candidate for regional anesthesia using the AO spine classification system [6]. The patient received a spinal block using an alternative logrolling maneuver and underwent an emergency CS with uterine repair without surgical or anesthesia complications. A viable infant was delivered. The patient was discharged two days later.

## Case presentation

A 27-year-old G3P2002 female at 37+6 weeks of gestation presented to the emergency department following a high-speed motor vehicle accident with significant abdominal pain, irregular contractions, and without ruptured membranes. Antenatal care was limited due to late entry of care at 32+0 weeks and was complicated by rubella non-immune status, underinsurance, and food insecurity. No significant past medical history was documented. Past obstetric history included two spontaneous vaginal deliveries, gravida 1 at 41+0 weeks with a viable female infant and gravida 2 at 39+0 weeks with a viable female infant. Initial prenatal workup revealed Rh+ blood type.

On arrival, vital signs and fetal heart tones were evaluated. She presented with minimal variability and late decelerations on electronic fetal monitoring and contractions every 1-2 minutes on the tocometer, classifying as a persistent Category II tracing. She was tachycardic and normotensive. Labs were significant for transaminitis with low suspicion of a hypertensive or ischemic etiology (Hb 10.7 g/dL, WBC 14.5 ×10³/µL, Plt 208 ×10³/µL). Resuscitative measures were initiated with a fluid bolus of one liter of lactated Ringer's with maintenance fluids as needed and repositioning as able, given the complex state. Coagulative panels were ordered and are pending. Results later demonstrated prothrombin time (PT) 10.2 s, international normalized ratio (INR) 1.0, activated partial thromboplastin time (aPTT) 25.1 s, and fibrinogen 486 mg/dL. Physical exam revealed a positive seatbelt sign in the lower abdomen and severe pain with movement of the lower extremities. CT imaging confirmed a right first rib fracture, right apical pneumothorax, and L2-4 right transverse processes fractures. She was stable for cesarean section, and emergent delivery was indicated.

**Figure 1:**
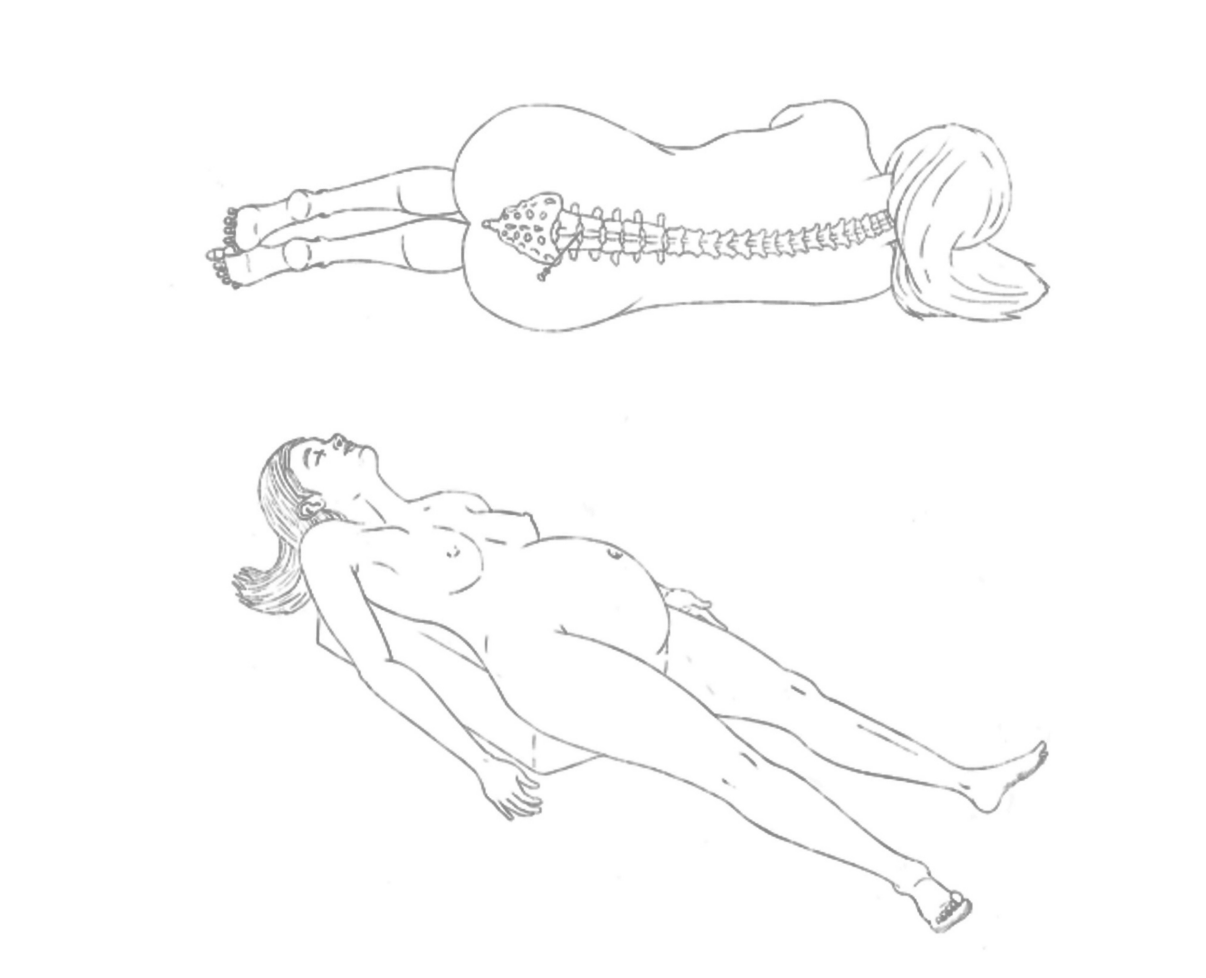
Hand-drawn depiction of the logrolling maneuver used to apply spinal anesthesia Image credits: Stewart Carter

**Figure 2:**
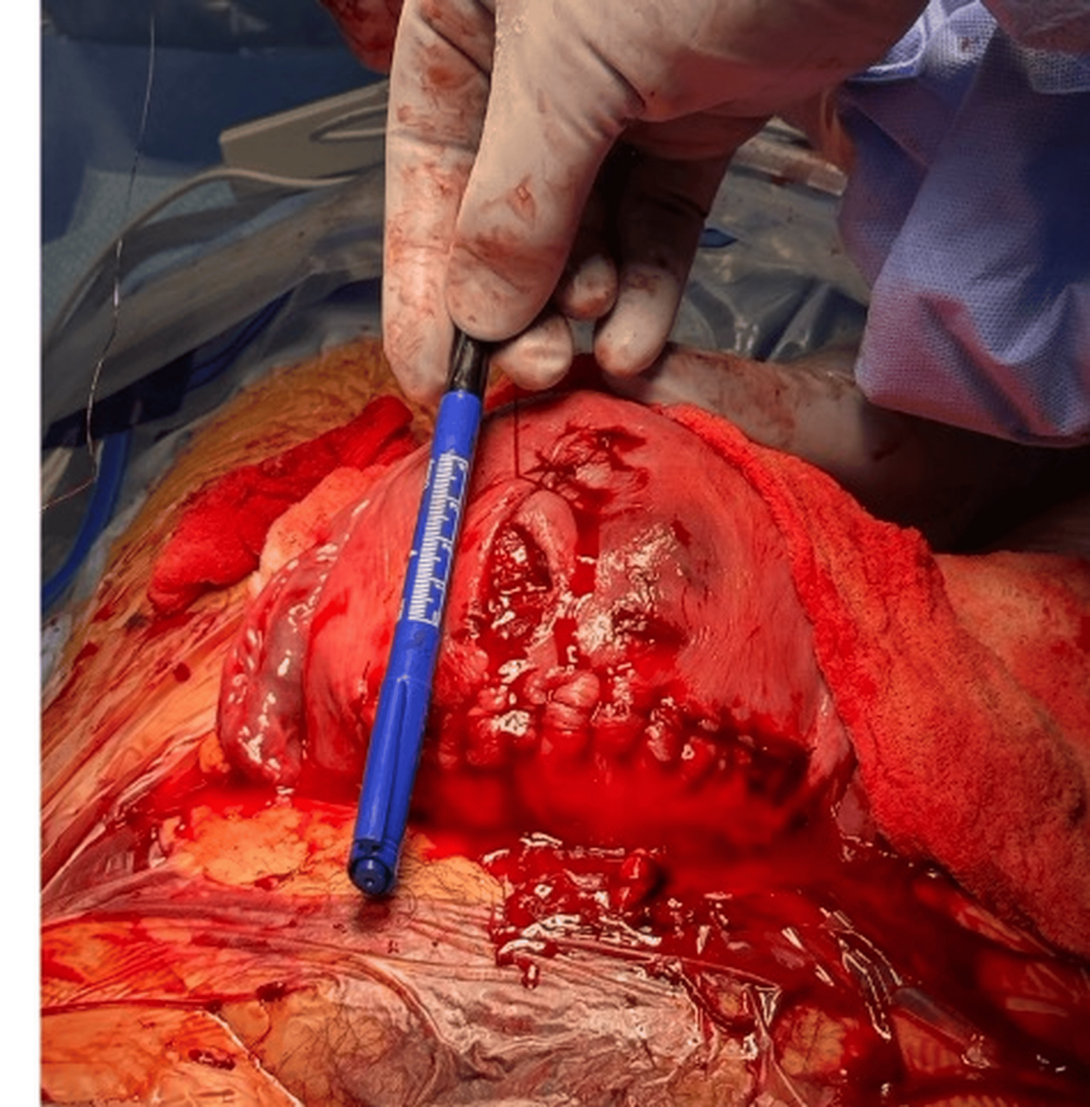
Uterine rupture post-repair, height measurement

**Figure 3:**
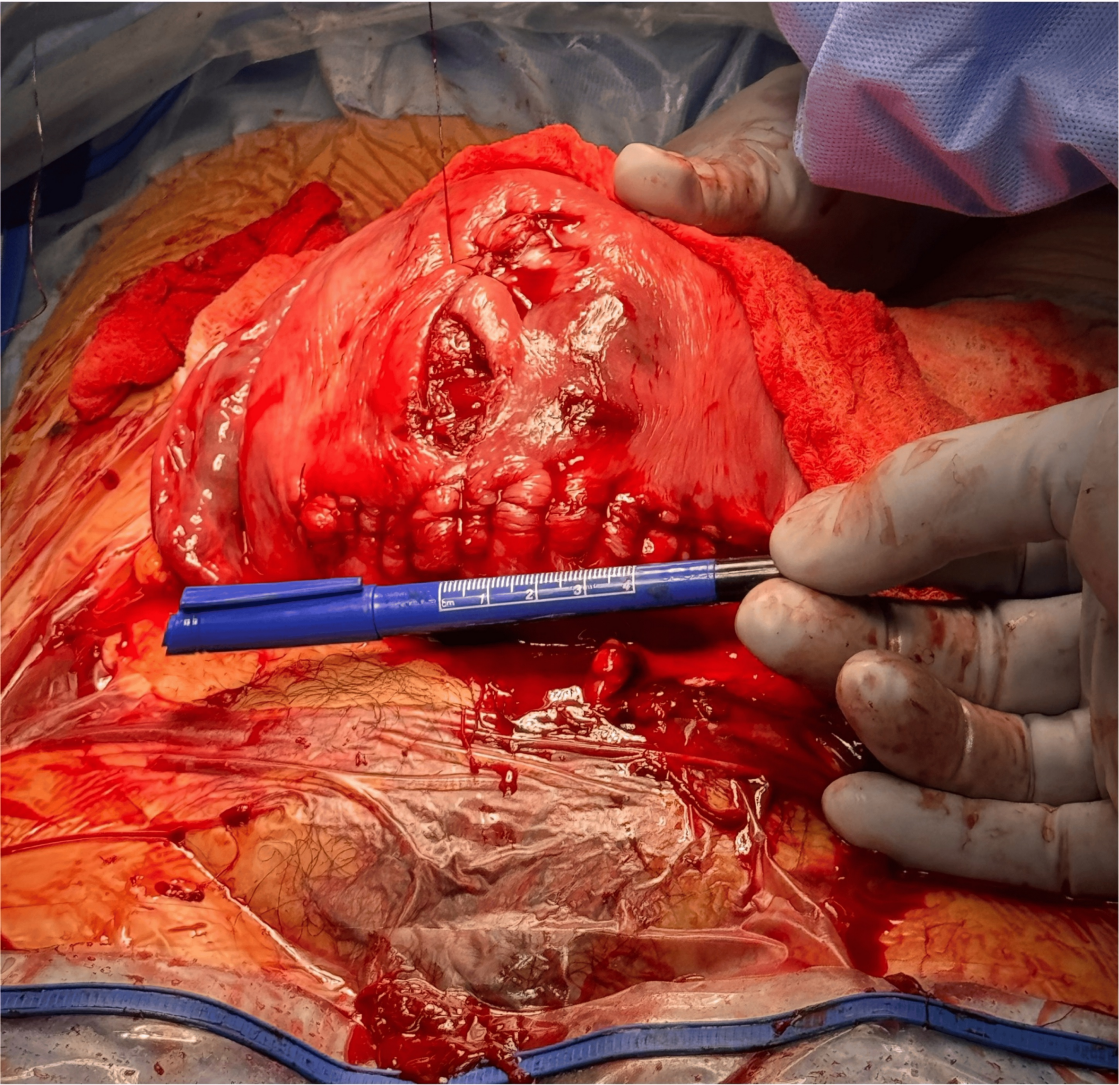
Uterine rupture post-repair, length measurement

Based on the AO spine classification system [6], the patient’s spine was stable, and the anesthesiology team implemented a logrolling maneuver to place the patient in a lateral decubitus position and administered spinal anesthesia. The patient was then gently rolled back to the supine position with a left lateral tilt (Figure 1). Upon entry into the abdomen, a 6 x 2.5cm vertical midline uterine rupture was observed superior to the line of hysterotomy and included the serosa and underlying myometrium (Figures 2, 3).

A cesarean section was performed, and a viable female infant was delivered. Apgar scores were 8/8. A hematoma was observed intraoperatively and was reduced. The uterus and paracolic gutters were cleared of debris. The uterus was re-examined and reinserted into the pelvis. The patient lost approximately 1000 cc of blood intraoperatively and required one unit of fresh frozen plasma and 1 gram of tranexamic acid.

Postoperatively, the patient was diagnosed with acute stress disorder and depressive mood symptoms. The patient was deemed stable and cleared for discharge on postpartum day two.

## Discussion

Blunt abdominal traumas increase the risk of uterine rupture and pose a major risk for increased fetomaternal morbidity and mortality [1,7]. Motor vehicle accidents are the leading cause of life-threatening traumas during pregnancy, and are associated with higher rates of preterm birth, surgical intervention, and intensive care [7,8]. Patients with unscarred uteri are at risk for maternal blood loss, hysterectomy, and greater maternal morbidity relative to scarred uteri [3]. Patients over the age of 30 are at an increased risk of uterine rupture, fetal mortality, and neurologic injury [3]. The morbidities were minimized in our patients’ and neonates’ cases, despite this patient having an unscarred uterus. Several factors, including age, location of uterine laceration, multiparity, and fetal age, as seen in this case, are associated with less severe patient outcomes.

This case highlights the multidisciplinary approach needed to manage anesthesia in the case of an obstetrical emergency with a stable spinal injury while minimizing postpartum complications. A broad literature search across two databases, PubMed and Embase, was performed. To our knowledge, this is the first case describing this anesthesia approach in the setting of obstetrical trauma. Spinal precautions, such as log rolling, are common and well-documented in instances of spinal instability in cases of pelvic, femoral, and spinal trauma. Current medical literature does not address spinal instability in obstetrical trauma. This identifies a major gap in the literature on obstetrical trauma, which is necessary to provide pregnant patients with optimal care. 

Colleagues in Turkey, Iran, and China have described log rolling and spinal block placement in the lateral decubitus position in non-traumatic cases [9-11]. According to Tan and Günaydın et al. (2014), the risks associated with lateral decubitus placement include, on average, an increase in the number of attempts at epidural insertion as well as paresthesia development. However, sensory blocks placed in a lateral decubitus position achieve a higher dose effect in a shorter time, which may be advantageous in the setting of trauma [9]. Postoperatively, there are no significant differences in hypotension, nausea, and vomiting outcomes [11].

Vaccaro et al. developed the AOSpine thoracolumbar spine injury classification system to create a simple, widely accepted, yet comprehensive stratification of spinal injuries classified by three injury patterns: compression (type A), tension band disruption (type B), and displacement/translation (type C) injuries [12]. Within the compression (type A) category, A0 is assigned to a clinically insignificant spinal fracture limited to the transverse and spinal processes. This patient’s spinal fracture was categorized as type A0, considering the fractures were limited to L2-4 transverse processes with no direct disruption to the spine. Therefore, the patient’s spine was deemed stable for spinal anesthesia, as opposed to general anesthesia. If the patient presented with a spinal fracture meeting the criteria of A4 or higher, general anesthesia would have been appropriate. A2 and A3 classifications require anesthesia-led comorbidity evaluation to guide selection of spinal or general anesthesia.

## Conclusions

Blunt abdominal trauma puts pregnant patients at risk for uterine rupture. Presentation of uterine rupture varies widely but should be included in the differential diagnosis for patients following abdominal trauma such as falls and motor vehicle accidents. This case illustrates a unique approach to anesthesia management in pregnant patients with unstable spines and further highlights the importance of assessing each patient’s needs.

## References

[1] Togioka BM, Tonismae T (2025). Uterine rupture. StatPearls [Internet].

[2] Al-Zirqi I, Stray-Pedersen B, Forsén L, Daltveit AK, Vangen S (2016). Uterine rupture: trends over 40 years. BJOG.

[3] Gibbins KJ, Weber T, Holmgren CM, Porter TF, Varner MW, Manuck TA (2015). Maternal and fetal morbidity associated with uterine rupture of the unscarred uterus. Am J Obstet Gynecol.

[4] Guise JM, Denman MA, Emeis C (2010). Vaginal birth after cesarean: new insights on maternal and neonatal outcomes. Obstet Gynecol.

[5] Vallejo MC, Kumaraswami S, Zakowski MI (2024). American Society of Anesthesiologists 2023 guidance on neurologic complications of neuraxial analgesia/anesthesia in obstetrics. Anesthesiology.

[6] (2026). AO Spine Classification Systems—the complete guides. https://www.aofoundation.org/spine.

[7] Sakamoto J, Michels C, Eisfelder B, Joshi N (2019). Trauma in pregnancy. Emerg Med Clin North Am.

[8] Ağaoğlu Z, Tanacan A, Haksever M (2024). Retrospective analysis of the indications, methods, and complications of pregnancy termination. Turk J Obstet Gynecol.

[9] Tan ED, Günaydın B (2014). Comparison of maternal and neonatal effects of combined spinal epidural anaesthesia in either the sitting or lateral position during elective cesarean section. Turk J Anaesthesiol Reanim.

[10] Atashkhoei S NB, Farzin H, Saeede M, Marandi PH, Hojjat P (2018). Effect of position during induction of spinal anaesthesia for caesarean section on maternal haemodynamic: randomised clinical trial. J Clin Diagn Res.

[11] Wen C, Xiang YY, Pang QY, Liu HL (2024). Effects of neuraxial anesthesia in sitting and lateral positions on maternal hemodynamics in cesarean section: a systematic review and meta-analysis. PLoS One.

[12] Vaccaro AR, Oner C, Kepler CK (2013). AOSpine thoracolumbar spine injury classification system. Fracture description, neurological status, and key modifiers. Spine (Phila Pa 1976).

